# Supplementary material for: Complex interactions in the life cycle of a simple parasite shape the evolution of virulence
Source: PLoS Pathog. 2025 Jun 30;21(6):e1013294. doi: 10.1371/journal.ppat.1013294 (PMC12225978; doi:10.1371/journal.ppat.1013294)
Supplement: Attachment — Submitted filename: Review_Silva_PlosPath.pdf [file ppat.1013294.s014.pdf]

Review of ‘Complex interactions in the life-cycle of a simple parasite shape the evolution of virulence’.

This study compares parasite life-history traits of the microsporidian, *Vavraia culicis*, selected for either early or later transmission in the mosquito host, *Anopheles gambiae*. The study finds that later transmission is associated with higher virulence, measured as survival, as well as higher levels of parasite growth. The study also measures effects of the parasite on host fecundity, finding that ancestor parasites had a greater reduction on fecundity than both early and late transmission parasites.

The study is nice and the many different results make it possible to have a real idea of how the selection regime affected parasite life-history. However, the results, as presented are quite complicated and difficult to follow and it is not clear whether some results are being obscured by the choice of analysis. I am not sure if you have the data for spore production from larvae and pupae; this data would be interesting to include as spores from these dead hosts would definitely be released into the aquatic environment and thus transmission more likely.

Overall, the results go in different directions. Sometimes the effect of late parasites are worse than early and vice versa. Is there a way to get a composite measure of virulence across life-stages? As you have each step of the parasite life-cycle it might be possible to combine all the traits as in de Roode et al, PNAS, 2008 to get a full measure of parasite fitness?

The experiment compares the traits of parasites selected for early or late transmission with the ancestral/stock population. Many of the infection traits measured in hosts compare the effects of ‘early’, ‘late’ and ‘stock’ parasites, but doesn’t include uninfected controls. The only reference to uninfected controls is the measure of fecundity. Why do you not include uninfected controls in the other measures of virulence? The uninfected controls don’t need to be included in every analysis, but it would be helpful to state somewhere what you may expect, for instance, in the absence of infection would all mosquitoes be alive after 30 days?

Abstract:
Lines 37 – 38: Your results do not show a shift to earlier reproduction if infected with the late parasites only. There is no regime * day interaction.

Lines 37 – 38: I wouldn’t discuss results from another study in the Abstract.

Introduction:
Line 50: It is not clear to me why for per parasite pathogenicity virulence is necessarily independent of growth. Surely more parasite would mean more toxins.

Methods:

Lines 348-350: Please provide more information about how the *V. culicis* is maintained. How frequently are dead hosts collected to create an inocula for new infections? How often do

you change host species? Are these infections controlled in the same way with 10000 spores per larva?

Line 351: Describe the infection protocol here.

Lines 377-392: The description of the experiments measuring the responses to selection are not clear. How were the spore solutions stored prior to the infections? Were all assays done at the same time? How many larvae were infected with each parasite from each selection regime and replicate? It looks like a certain number of adults were allocated to measure fecundity and spore rate -how was this decided? How many females were culled each day to measure spore rate?

Line 398: What do you mean 'their diagnosis'?

Lines 402-404: please describe the methods here rather than referring the reader to another article.

Line 407-409: Was the proportion of non-egg laying females just among the 100 isolated in each regime?

Lines 411 – 414: Why not just measure the fecundity of females that had laid eggs and compare with uninfected females?

Lines 413-415: This is the first time days 10 and 15 are mentioned. It is stated in lines 414-415 this is days post-emergence. This should be stated earlier on in the methods.

Line 416: This is the proportion of all individuals in the experiment who died as larvae or pupae?

Lines 419 – 420: Why don't you just measure time to pupation? It is not clear why you split this trait into early or late.

Line 425 – 426: The term 'spore rate' alone is unclear. You should add production into the term 'spore production rate', or 'rate of spore production in infected adult females'. Also, why do you only concentrate on adult females and not also larvae and pupae?

Results

Lines 117- 118: You also measure the effects of the parasite on other host traits. This is a huge strength, why not just state that you have multiple measures of virulence?

Lines 120-122: This is confusing. Describe in more detail here what you mean?

Lines 130 – 133 & Figure 2c: It is a bit confusing to present the ln hazard ratios. Presenting the untransformed hazard ratios would be simpler to interpret.

Lines 135 – 136: The Appendix table just presents the model. More information about the analyses –package used, whether you simplified the model etc would be helpful.

Lines 142-147: It is not clear why you do these post-hoc comparisons when the interaction was not significant.

Line 150 - 152: Higher fecundity for females emerged on day 10 could be fecundity compensation.

Line 167 - 169: State here again this is in adult females only and exactly what you mean by spore rate (or re-define).

Lines 432-440 and lines 179 – 199: These analyses are really confusing to follow and it is not clear whether by doing the analyses in certain ways obscures what is going on.

1. It is not clear to what extent measuring exploitation as mean spore load from days 15 – 18 excludes data from each replicate. Why not include densities across all individuals? Please include numbers of individuals from each replicate included in these calculations and justify why done in this way.

2. It is not clear how the measure of per parasite pathogenicity is independent of parasite growth. Even if due to toxin production won't more parasite translate into more toxins? Why aren't the ancestor parasites included in this analysis. Why is day included in this analysis (Table S4) if spore density taken as the mean from days 15 – 18 and virulence ln hazard ratio (one measure per replicate? It is not clear to me how the stats in Table S4 link to the Figure 4b. Although figure 4b shows no relationship between spore density and virulence within each regime, across regimes it looks like there is a positive relationship.

Lines 197 – 198: This analysis on 'infectiousness' should be described in the 'Statistical Analyses' section. Also, this should be called transmission potential. Infectiousness would be the proportion of hosts that actually become infected. I would though be cautious about this as these dead females would have to die on water for the parasite to complete its life-cycle. Unless of course there is a link between spore number and numbers deposed on eggs. Do you know anything about this?

Discussion

The discussion is generally well written and interesting to read with an interesting discussion about factors influencing virulence evolution.

Line 291- 293: You could develop this discussion about fecundity compensation.

Lines 324 – 336: This last paragraph is a bit unrelated to the rest of the whole study. I don't think it is necessary.

Line 331: What do you expect wild mosquito populations densities to be?

Lines 331-334: Why would you expect mechanisms associated with *Plasmodium* suppression to trade-off with development? Is it costly?
